# Identifying causative mechanisms linking early-life stress to psycho-cardio-metabolic multi-morbidity: The EarlyCause project

**DOI:** 10.1371/journal.pone.0245475

**Published:** 2021-01-21

**Authors:** Nicole Mariani, Alessandra Borsini, Charlotte A. M. Cecil, Janine F. Felix, Sylvain Sebert, Annamaria Cattaneo, Esther Walton, Yuri Milaneschi, Guy Cochrane, Clara Amid, Jeena Rajan, Juliette Giacobbe, Yolanda Sanz, Ana Agustí, Tania Sorg, Yann Herault, Jouko Miettunen, Priyanka Parmar, Nadia Cattane, Vincent Jaddoe, Jyrki Lötjönen, Carme Buisan, Miguel A. González Ballester, Gemma Piella, Josep L. Gelpi, Femke Lamers, Brenda W. J. H. Penninx, Henning Tiemeier, Malte von Tottleben, Rainer Thiel, Katharina F. Heil, Marjo-Riitta Järvelin, Carmine Pariante, Isabelle M. Mansuy, Karim Lekadir

**Affiliations:** 1 Department of Psychological Medicine, Stress, Psychiatry and Immunology Laboratory, Institute of Psychiatry, Psychology & Neuroscience, King’s College London, London, United Kingdom; 2 Department of Epidemiology, Erasmus MC, University Medical Center Rotterdam, Rotterdam, The Netherlands; 3 Department of Child and Adolescent Psychiatry, Erasmus MC, University Medical Center Rotterdam, Rotterdam, The Netherlands; 4 Generation R Study Group, Erasmus MC, University Medical Center Rotterdam, Rotterdam, The Netherlands; 5 Department of Pediatrics, Erasmus MC, University Medical Center Rotterdam, Rotterdam, The Netherlands; 6 Faculty of Medicine, Center for Life Course Health Research, University of Oulu, Oulu, Finland; 7 Medical Research Council Integrative Epidemiology Unit, Bristol Medical School, University of Bristol, Bristol, United Kingdom; 8 Faculty of Medicine, Department of Metabolism, Digestion and Reproduction, Genomic Medicine, Imperial College London, London, United Kingdom; 9 IRCCS Istituto Centro San Giovanni di Dio Fatebenefratelli, Biological Psychiatry Laboratory, Brescia, Italy; 10 Department of Pharmacological and Biomolecular Sciences, University of Milan, Milan, Italy; 11 Department of Psychology, University of Bath, Bath, United Kingdom; 12 Department of Psychiatry, Amsterdam UMC/Vrije Universiteit & GGZinGeest, Amsterdam Public Health and Amsterdam Neuroscience Research Institutes, Amsterdam, The Netherlands; 13 European Molecular Biology Laboratory, European Bioinformatics Institute, Wellcome Genome Campus, Hinxton, Cambridge, United Kingdom; 14 Department of Viroscience, Erasmus Medical Center, Rotterdam, Netherlands; 15 Microbial Ecology, Nutrition and Health Research Group, Institute of Agrochemistry and Food Technology, National Research Council (IATA-CSIC), Valencia, Spain; 16 Centre Européen de Recherche en Biologie et Médicine, Institut de Génétique et de Biologie Moléculaire et Cellulaire, PHENOMIN-ICS, Université de Strasbourg, CNRS, INSERM, Strasbourg, France; 17 Medical Research Center Oulu, Oulu University Hospital and University of Oulu, Oulu, Finland; 18 Department of Information and Communication Technologies, Universitat Pompeu Fabra, Barcelona, Spain; 19 Catalan Institution for Research and Advanced Studies (ICREA), Barcelona, Spain; 20 Department of Biochemistry and Molecular Biomedicine, Universitat de Barcelona, Barcelona, Spain; 21 Department of Social and Behavioral Science, Harvard T.H. Chan School of Public Health, Boston, Massachusetts, United States of America; 22 Empirica Communication and Technology Research, Bonn, Germany; 23 Departament de Matemàtiques i Informàtica, Universitat de Barcelona, Barcelona, Spain; 24 Department of Epidemiology and Biostatistics, MRC-PHE Centre for Environment and Health, School of Public Health, Imperial College London, London, United Kingdom; 25 Unit of Primary Health Care, Oulu University Hospital, OYS, Oulu, Finland; 26 Department of Life Sciences, College of Health and Life Sciences, Brunel University London, London, United Kingdom; 27 Medical Faculty of the University of Zürich and Department of Health Science and Technology of the ETH Zürich, Laboratory of Neuroepigenetics, Brain Research Institute, Zürich Neuroscience Center, Zürich, Switzerland; University of Minnesota, UNITED STATES

## Abstract

**Introduction:**

Depression, cardiovascular diseases and diabetes are among the major non-communicable diseases, leading to significant disability and mortality worldwide. These diseases may share environmental and genetic determinants associated with multimorbid patterns. Stressful early-life events are among the primary factors associated with the development of mental and physical diseases. However, possible causative mechanisms linking early life stress (ELS) with psycho-cardio-metabolic (PCM) multi-morbidity are not well understood. This prevents a full understanding of causal pathways towards the shared risk of these diseases and the development of coordinated preventive and therapeutic interventions.

**Methods and analysis:**

This paper describes the study protocol for EarlyCause, a large-scale and inter-disciplinary research project funded by the European Union’s Horizon 2020 research and innovation programme. The project takes advantage of human longitudinal birth cohort data, animal studies and cellular models to test the hypothesis of shared mechanisms and molecular pathways by which ELS shapes an individual’s physical and mental health in adulthood. The study will research in detail how ELS converts into biological signals embedded simultaneously or sequentially in the brain, the cardiovascular and metabolic systems. The research will mainly focus on four biological processes including possible alterations of the epigenome, neuroendocrine system, inflammatome, and the gut microbiome. Life-course models will integrate the role of modifying factors as sex, socioeconomics, and lifestyle with the goal to better identify groups at risk as well as inform promising strategies to reverse the possible mechanisms and/or reduce the impact of ELS on multi-morbidity development in high-risk individuals. These strategies will help better manage the impact of multi-morbidity on human health and the associated risk.

## 1. Introduction

### 1.1 Early life stress and psycho-cardio-metabolic multi-morbidity

The World Health Organisation has identified mental disorders, including depression, cardiovascular diseases and diabetes among the six major non-communicable diseases [1]. Individually, each of these groups of diseases represents a burden at the individual and population level. Depression alone is the single largest contributor to global disability, accounting for 12% of total years lived with disability [2] with more than 300 million individuals affected per year. Cardiovascular diseases (CVDs) remain the prime cause of mortality worldwide, accounting for about a third of annual deaths [3]. Finally, type 2 diabetes and related metabolic dysfunctions, including obesity, are a major public health challenge, with an average prevalence of over 8% in the general population [4]. In addition to their separate complexity, existing research has shown important multi-morbidity between these diseases, where multi-morbidity is defined as the co-occurrence of two or more chronic conditions [5]. Epidemiological studies have indeed shown that for example patients experiencing depression are more likely to have comorbid CVD [6], type 2 diabetes [7], or both [8]. However, the specific causative mechanisms leading to psycho-cardio-metabolic (PCM) multi-morbidity are not well understood, which limits the development of effective preventive and therapeutic measures.

Recent evidence suggests that many mental and physical conditions find their origins in exposure to stress early in life, otherwise defined as early-life-stress (ELS) [9]. ELS can be both prenatal, such as exposure to clinically-significant depression *in utero*, and postnatal, such as emotional, physical and sexual abuse or neglect in childhood, parental psychopathology and separation, prepubertal bullying, as well as victimisation or violence by peers [10]. Growing evidence has supported an association between ELS (both prenatal and postnatal) and the development of the PCM conditions. Specifically, patients with a history of ELS have higher vulnerability for depression [11], and higher risk of developing cardiovascular disease [12], obesity [13] and type 2 diabetes [14] later in life. Prenatally, the overarching hypothesis is that the maternal stress response is passed to the fetus, via stress hormones crossing the placenta, which affects subsequent brain and physical development of the fetus and newborn [15]. During childhood, exposure to excessive levels of stress early in life can cause several biological alterations which can ultimately favour the development of PCM multi-morbidity [16]. As suggested by Barker’s work on the developmental origins of chronic diseases, including PCM conditions [17], the exact predictors of the development of these diseases are to be linked with variations of key systems during the developmental stage. Examples of key biological system alterations due to responses to stress include hypothalamic-pituitary-adrenal (HPA) axis changes as a response to stress [18], changes in the inflammatory response [19], microbiome dysbiosis [20,21] and overall bio-psycho-social axis dysfunction [22]. While ELS has also been linked to resilience in adulthood [23], our research will focus on understanding the mechanisms leading to the negative consequences, in particular PCM multi-morbidity. Considering that the prevalence of ELS, both *in utero* and postnatally, whether mild or severe, has reached alarming heights [15], this area of research is essential for future disease prevention and health promotion.

The objective of this paper is to present the EarlyCause project, a large-scale interdisciplinary research that aims to infer evidence for causative mechanisms linking pre- and postnatal ELS to PCM multi-morbidity, even decades after the exposure itself has ceased. To explain this enduring effect, EarlyCause will seek to identify both biological mediators and environmental moderators. With regards to biological mediators, the hypothesis is that the enduring effects of ELS may reflect in part a “biologically embedding”, whereby ELS alters biological development and function in a way that engenders latent vulnerability for poor health outcomes and increased susceptibility. This is supported by the identification of numerous biological correlates of ELS, and previously mentioned, including neuroendocrine dysregulation, heightened inflammatory response, changes in gut microbiota composition, and, more recently, alterations to the epigenome. The ambition of EarlyCause is to go well beyond reductionist approaches which have traditionally investigated new biomedical knowledge while treating disease classes, biological scales and temporal domains (*e*.*g*. childhood vs. adulthood) separately. We will thus investigate more holistic models of the causative pathways by building on the experience of our consortium in advanced methods such as Mendelian randomisation, structural equation modelling, multi-omics, and machine/deep learning. We will detail below EarlyCause objectives and hypotheses, as well as the methods that will be implemented to test these hypotheses (see Method section).

Furthermore, the EarlyCause consortium fully understands that given the prevalence of ELS affecting millions of mothers and children, important efforts need to be dedicated from day one to maximising the clinical and socioeconomic impacts. As one of its core objectives, EarlyCause will ensure that each impact is adequately assessed from clinical and non-clinical perspectives, as to allow future exploitation of the innovation outputs. This work will be done while taking into account the current gaps and limitations due to the single-disease frameworks that have dominated for a very long-time research, biotech innovation and healthcare.

### 1.2 Rationale and overview of the EarlyCause project

EarlyCause is the product of a collaboration between 14 participating institutions across Europe (Table 1) and is supported by the European Union’s Horizon 2020 research and innovation programme (SC1-BHC-01-2019).

**Table 1:** Participating institutions in the EarlyCause project.

Participant no.	Participant organisation name	Acronym	Country
1 (Coordinator)	Universitat de Barcelona	UB	ES
2	European Molecular Biology Laboratory	EMBL	DE
3	Erasmus Medical Centre Rotterdam	EMC	NL
4	University of Zurich	UZH	CH
5	King’s College London	KCL	UK
6	Consejo Superior de Investigaciones Cientificas	CSIC	ES
7	Centre Européen de Recherche en Biologie et Médicine	CERBM	FR
8	University of Oulu	OULU	FI
9	Fatebenefratelli Institute	IRCCS	IT
10	University of Bath	UOB	UK
11	VU Medical Centre, Amsterdam	VUMC	NL
12	Empirica Communication and Technology Research	EMP	DE
13	Combinostics Oy	COMBI	FI
14	Universitat Pompeu Fabra	UPF	ES

EarlyCause will investigate the hypothesis that ELS, as a risk factor for depressive, cardiovascular and metabolic disorders individually, is a cause of multi-morbidity between these conditions. From a biological point of view, the main hypothesis is that ELS activates a chain of events leading to cellular, molecular, epigenetic and microbial changes from the norm. This causative chain would ultimately trigger specific cellular and tissue phenotypes and comorbid pathological traits in the mental, cardiovascular and metabolic domains.

To this end, EarlyCause’s overarching concept is to build upon a unique repertoire of longitudinal data in humans across the lifespan and conduct mechanistic studies in established animal and cellular models to:

- Identify the causal mechanisms linking exposure to ELS to the risk of multi-morbid symptoms across life;
- Delineate the potential molecular mechanisms underlying these causal associations;
- Discover new biomarkers tapping into multiple biological domains;
- Build integrative computational models and proof-of-concept tools for multi-morbidity assessment.

The project will focus on four candidate families of biological pathways that have been linked to ELS, specifically:

1. **Epigenetic alterations** are a presumed link between stress exposure and phenotypes. Clear associations between early-life adverse exposure and epigenetic processes (e.g. DNA methylation) and between these epigenetic modifications and later health outcomes have been shown both in humans [24–26] and mouse models [27].
2. **HPA changes** have been associated with ELS exposure [18]. Molecular components of the HPA axis provide a relay chain across the body from the brain to the periphery, and some of the final products (glucocorticoid hormones) are potent regulators of glucose and lipid metabolism. Thus, this represents a central candidate mechanistic player in the aetiology of multi-morbid symptoms.
3. **Inflammatory pathways** are a form of cellular response to ELS [19] reflecting activation of white blood cells in the circulation and peripheral tissues such as the spleen, lymph nodes and adipose tissue. Inflammatory components may have profound effects on the cardiovascular system, endothelial accumulation and activation of plaques, and adipose tissue metabolism, whose dysfunction has been associated with stress-related diseases including depression, cardiovascular disease and diabetes.
4. **Gut microbiome** is a major contributor to health and disease [20,21], which plays a key role in modulating immune, neuroendocrine and behavioural responses to ELS, as proven mainly in mouse models [28].

In addition to these biological factors, EarlyCause will test the potential moderating role of key factors such as sex, socioeconomics, and lifestyle in the association between ELS and multi-morbidity development. Evidence for causality, mediation and moderation will be used to identify potential targets for intervention acting on the causative mechanisms to reduce the impact of ELS on multi-morbidity development in high-risk individuals.

Specifically, using longitudinal human data, as well as animal and cellular models we aim to address the following hypotheses (Table 2).

**Table 2:** Table of hypotheses for longitudinal human data, animal and cellular models.

Model	Hypotheses
Longitudinal human data (section 2.1)	Hypothesis 1: Early-life stress, including sexual, physical, psychological abuse and/or neglect in early life, is a causal factor in the development of PCM multi-morbidity. Specifically, ELS is a shared risk factor for pre-clinical psychological and cardiometabolic symptoms in childhood and PCM multi-morbidity in adulthood;
	Hypothesis 2: Specific alterations in DNA methylation inflammation, neuroendocrine function, and/or the gut microbiota mediate the ELS effects on PCM multi-morbidity;
	Hypothesis 3: The association between ELS and PCM is modifiable by lifestyle factors. We hypothesise positive moderation (prevention) by physical activity, dietary factors and sleep. In contrast, we hypothesise that the association is exacerbated by smoking and alcohol consumption;
	Hypothesis 4: PCM multi-morbidity is partly heritable and the individual genetic determinants of depression, and cardiometabolic diseases form a joint genetic factor of PCM symptoms in child- and adulthood.
Animal model (section 2.2)	Hypothesis 1: Exposure to pre- and postnatal stress induces behavioural, cardiovascular and metabolic changes across adulthood in mice;
	Hypothesis 2: The effect of pre- and postnatal stress on the above outcomes (Hypothesis 1) is mediated by similar epigenetic and molecular changes associated with PCM multi-morbidity symptoms previously identified in the human model.
Cellular model (section 2.3)	Hypothesis 1: Exposure of cells from the brain and peripheral organs to stress-related insults (cortisol and cytokines) induces cellular changes relevant to PCM multi-morbidity;
	Hypothesis 2: Mechanisms underlying the effect of the stress-related insults on the aforementioned cell types (Hypothesis 1) are mediated by molecular and gene expression changes previously identified both in the human and animal models.

## 2. Methods

**Fig 1:**
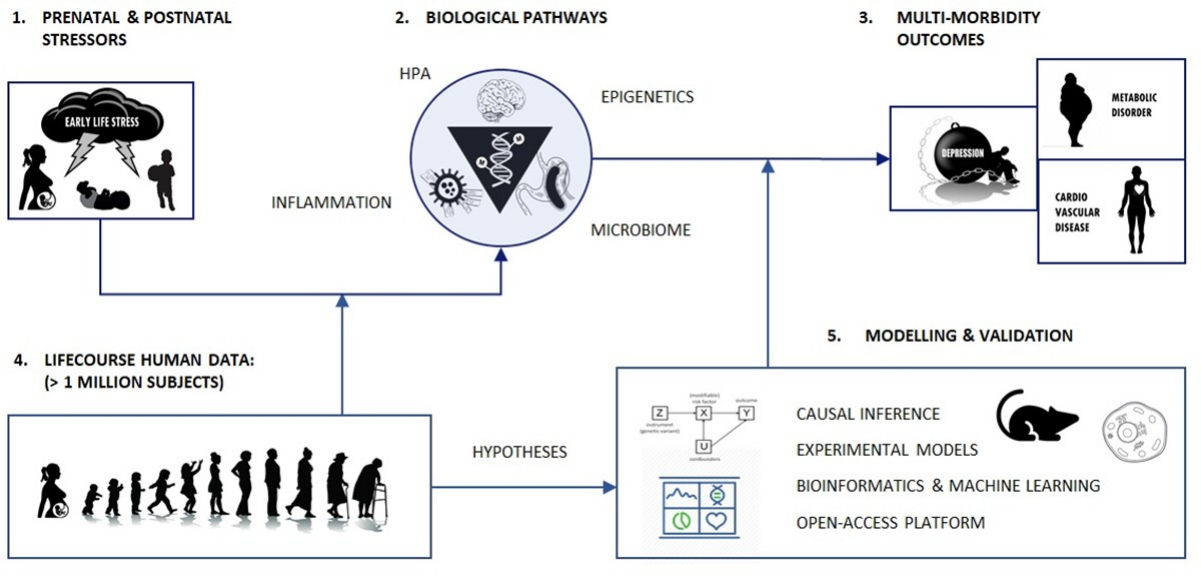
Schematic overview of the EarlyCause project, that will study the link between ELS (1) and multi-morbidity (3), as mediated by biological pathways (2). Large-scale life course human data (4), as well as experimental and computational models, will be used to identify and validate the causative mechanisms.

EarlyCause’s methodology is divided into multiple steps. As shown in *Fig 1*, the study protocol aims to triangulate evidence based on (1) longitudinal human data, (2) animal and cellular models, as well as (3) computational bioinformatics and machine learning methods. Concretely, hypotheses on associations between ELS and PCM outcomes, as well as on potential biological mediators and modifiers, will be generated from the longitudinal human data, and then translated into experimental designs to be validated in the animal and cellular models, which will be leveraged to identify the relevant causative mechanisms and molecular pathways (implementation details are provided in Sections 2.1 & 2.2).

**Fig 2:**
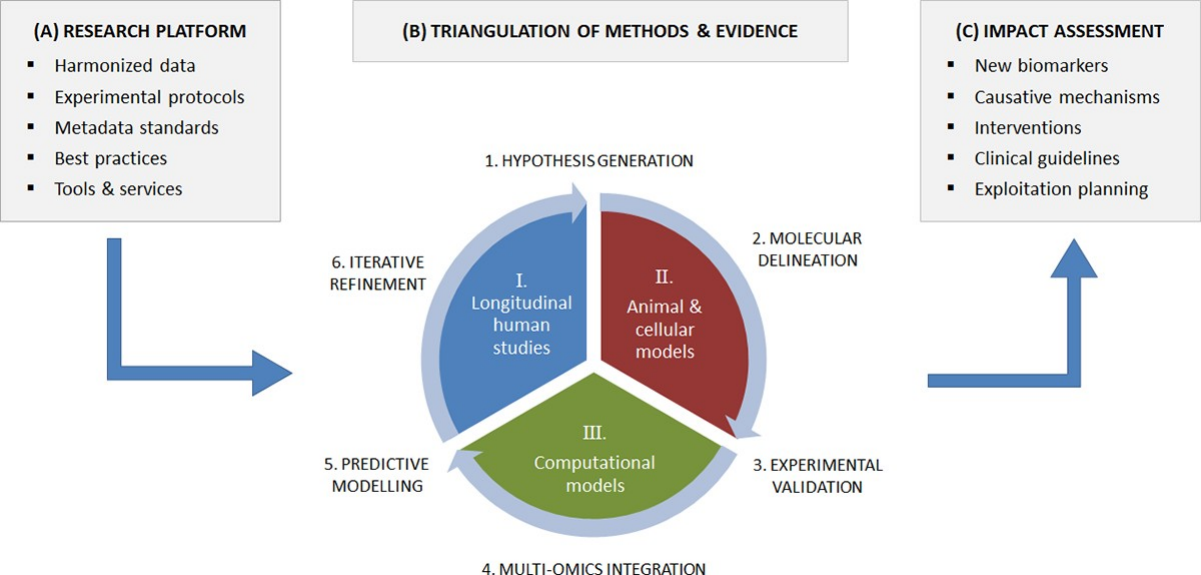
Overall methodology of the EarlyCause project, including (A) research platform of harmonised data from existing resources, research protocols and best practices; (B) triangulation approach, which will capitalise on the complementary strengths of epidemiological and genetic methods in humans, experimental animal and cellular models, and in silico data integration pipelines; and (C) expected results such as new biomarkers and clinical knowledge.

EarlyCause will implement a ‘triangulation’ approach, which will capitalise on the complementary strengths of epidemiological and genetic methods in humans, experimental animal and cellular models, and *in silico* data integration pipelines, as shown in *Fig 2*. This will enable us to iteratively and dynamically:

- Apply association analyses and latent modelling to large-scale longitudinal human data on ELS, resulting in the identification of potential new candidate biomarkers of PCM multi-morbidity, as well as novel hypotheses on underlying causative mechanisms;
- Apply causal inference methods, including structural equation modelling, Mendelian randomisation, and molecular mediation, to infer the causal relationships between ELS, biological mediators and the multimorbid outcomes;
- Validate the mechanisms and identify the associated molecular pathways in pre- and postnatal animal models, using established cellular models of stress;
- Integrate the identified determinants and molecular markers of ELS into computational models of multi-morbidity across the life span, and design a proof-of-concept decision support tool for PCM multi-morbidity risk assessment, by extending an existing single-disease e-health tool commercialised by EarlyCause partner COMBI (*i*.*e*., from the DSF^®^: *Disease State Fingerprint [29,30]* to the MSF: *Multi-morbidity State Fingerprint*).

### 2.1 Longitudinal human data

#### a. Hypothesis-generating analyses of biological markers & environmental moderators

We will leverage harmonized data from a large set of human studies to examine the relationship between ELS and multi-morbidity across the lifespan, identify potential molecular markers and quantify the protective vs. exacerbating role of modifiable lifestyle factors. These datasets together span from pregnancy to old age, including the well-known Avon Longitudinal Study of Parents and Children (ALSPAC), Generation R Study (GenR), Northern Finland Birth Cohorts (NFBC), UK Biobank, Rotterdam Study, and the Netherlands Study of Depression and Anxiety (NESDA).

Note that other prominent birth cohorts exist outside of Europe, such as the well-known Dunedin Study [31], which may be considered for inclusion in later stages of the project if there is a need to increase the sample size. While the analyses in the child cohorts (e.g., Generation R, ALSPAC) will focus on life events and circumstances experienced in childhood (up to age 10), later life events and circumstances—experienced in adolescences and adulthood—will be taken into account in the adult cohort analysis (e.g., NFBC, Rotterdam Study, NESDA). In particular, we aim to assess how ELS associates with later PCM multimorbidity by:

- adjusting for the continuity of life events (i.e., treating later adverse life events as covariates, allowing us to assess the direct and unique contribution of early exposure to life events);
- studying the mediating effect of later life events (i.e., treating later events as a continuum of earlier risk exposures). In these analyses, we are particularly interested in the mediating effects of educational attainment, own and parental socioeconomic position (including income), and substance (ab-)use, measured at a time when the study participants are in their teenage ages or later.

**Fig 3:**
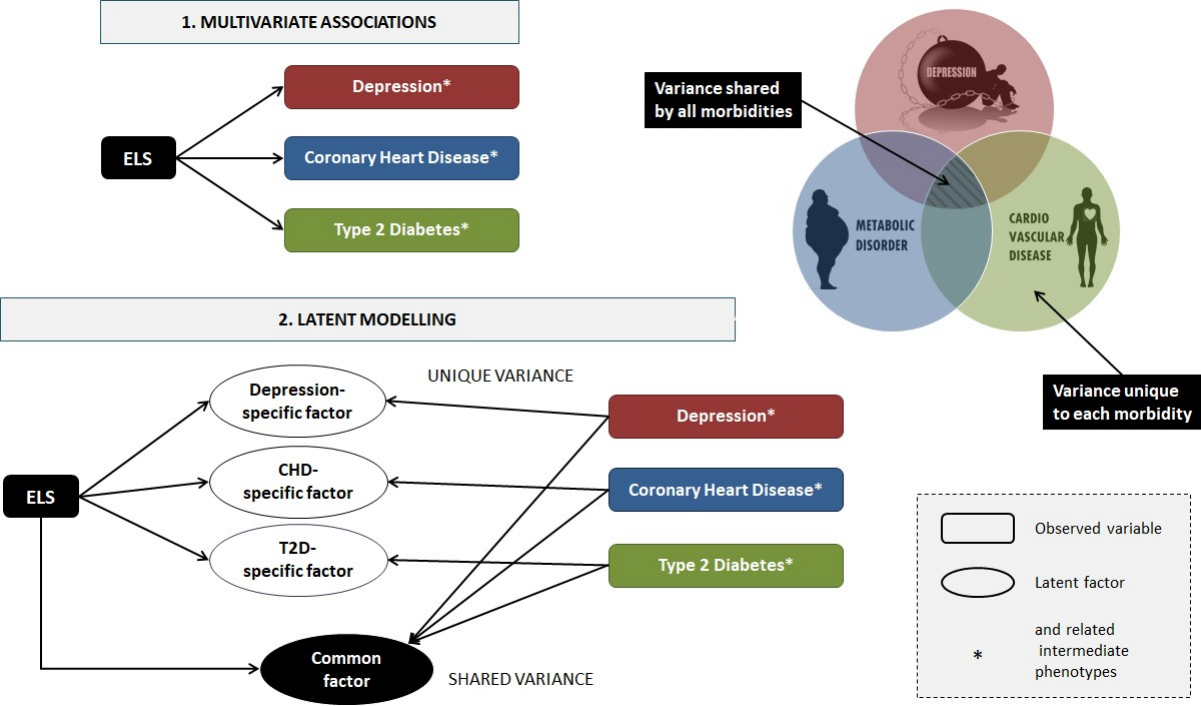
Differences between (1) simple multivariate analysis and (2) latent modelling of psycho-cardio-metabolic multi-morbidity implemented in EarlyCause.

This will allow us to assess the degree to which early life events are unique risk factors for PCM multimorbidity or act through a continuum of risk exposures that starts in childhood but carries on into adulthood. Furthermore, we will make use of correlational multivariate analyses as well as novel latent modelling techniques to model the shared versus the unique contribution of ELS on multi-morbid outcomes (*Fig 3*). In these human studies, we will:

- Define the relationship between ELS and multi-morbidity across the lifespan, by tracking risk factors of cardiovascular and metabolic disorders in children and adolescents, and the influence of early life stressors on tracking patterns, drawing a rich dataset of clinical samples and cohort studies publicly available or through members and collaborators of the consortium. We are currently seeking additional scientists and groups interested in collaborating with us;
- Identify candidate biological predictors and mediators of ELS effects on multi-morbidity (epigenetic marks, neuroendocrine function, inflammation, gut microbiome);
- Quantify the protective or exacerbating role of modifiable lifestyle factors and behaviours, which include exercise, diet, sleep, smoking and alcohol use in the relationships of ELS with biological markers and PCM multi-morbidity;
- Provide hypotheses and candidate biomarkers that can be used for causal inference and mediation studies, as well as in animal and in vitro studies;

#### b. Causal inference and molecular mediation analyses

To investigate whether ELS represents a *causal* risk factor for PCM multi-morbidity, and to what extent biological factors identified in the hypothesis-generating analyses represent shared non-causal biomarkers of ELS and PCM multi-morbidity, or point towards causal mediating mechanisms, we will use multiple approaches. We will apply multiple methods (triangulation) such as Mendelian randomisation, genetic risk score methods and associated sensitivity analyses [32] to infer causality using population-based human genetic data. Genetic summary measures on ELS and multi-morbidity will be derived through a meta-analysis of genome-wide association study (GWAS) data on childhood maltreatment as well as on health outcomes (i.e. depression, type 2 diabetes, and coronary heart disease). More specifically, we will:

- Establish a catalogue of genetic instrumental variables for ELS by performing a GWAS-meta-analysis across studies of human cohorts involved in EarlyCause and, if possible, including further studies with relevant data;
- Infer the causal association between postnatal ELS and multi-morbidity development through Mendelian randomisation and by using both diagnostic criteria and pre-diagnostic correlates of multimorbid outcomes;
- Establish the molecular mediation of biological markers (DNA methylation, cortisol, inflammation, microbiome) linking ELS exposure to later PCM multi-morbidity.

### 2.2 Animal and cellular models

#### a. Modelling ELS in animals for causality assessment

**Fig 4:**
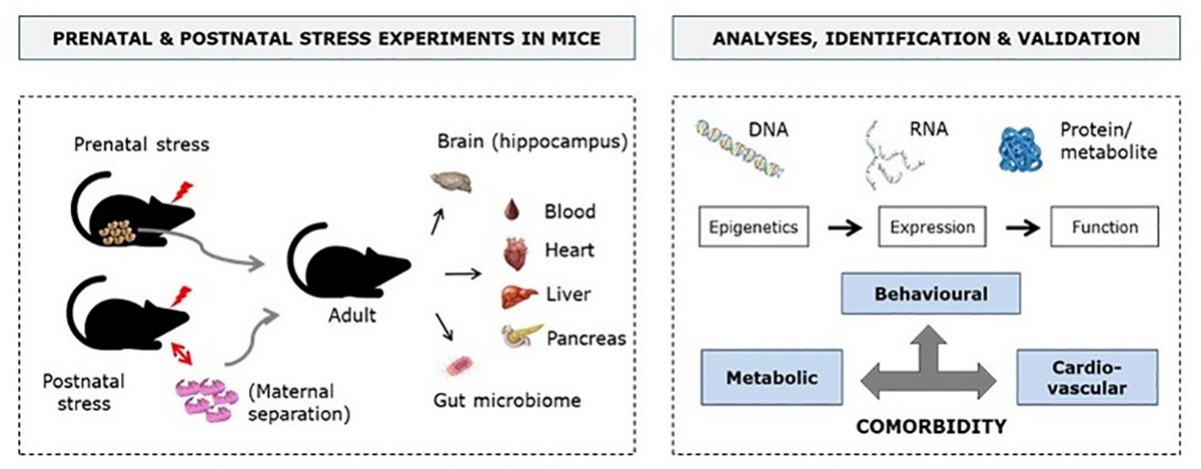
Overview of the experiments and analyses in rodents to identify molecular pathways linking ELS to PCM multi-morbidity.

We aim to exploit unique pre- and postnatal rodent models of stress [27,33] to identify ELS-associated molecular pathways causally linked to multi-morbid symptoms in adult life. For both models, we will determine which changes in the epigenome, transcriptome and proteome/metabolome are induced by ELS exposure across different tissues and biological fluids relevant for PCM symptoms, including brain, blood, heart, liver, pancreas and adipose tissue (*Fig 4*). The purpose will be to identify common and distinct epigenetic and neuroendocrine factors, immune markers and molecular pathways dysregulated by ELS in these tissues in the animal models. The observed alterations will be cross-validated with markers/pathways identified in humans via comparative analyses. Once epigenetic, neuroendocrine, immune and molecular alterations are identified, their potential reversibility by interventions such as environmental enrichment or pharmacological compounds will be assessed, based on previous knowledge that enriched life conditions have beneficial effects on brain and body functions. In a validation step, we will examine the causal involvement of relevant markers in the aetiology and expression of symptoms characteristic of depression, cardiovascular dysfunctions and metabolic alterations by experimental manipulations *in vivo*. Specifically, we will:

- Determine and quantify the impact of pre- and postnatal stress on behavioural, cardiovascular and metabolic functions in adulthood in rodents;
- Examine the effects of intervention and identify moderators relevant for humans;
- Identify epigenetic and molecular pathways associated with symptoms, and test causality *in vivo*;
- Assess the specific role of the human gut microbiome as a causative factor for the development of PCM multi-morbidity.

In particular, specific behavioural tests, including social interaction test, novel object recognition test, sucrose preference test, open field test, and novelty suppressed feeding test will be run. Basic metabolic parameters, stress-related endocrine markers (HPA axis) and cardiovascular functions will be assessed *in* and on tissues. Blood metabolic profiling will be conducted and components such as high/low-density lipoprotein (HDL/LDL), triglycerides, free fatty acids, glycerol, C reactive protein will be quantified with appropriate assays. Corticosterone (HPA-axis activation output) will be measured before and after an acute restraint stress. Blood pressure and heart rate will be measured by telemetry and tail-cuff, and glucose tolerance, insulin sensitivity, stress-induced glucose response with the Accu Check Aviva device. Cardiac functions and vascular tissue will be instead examined by echocardiography and electrocardiography, and 3D reconstruction of cardiac structure will be performed using high-resolution episcopic microscopy. Moreover, we will also examine the gut microbiome composition in association with ELS exposure in the animal models and the relationships with other molecular alterations, and compare the findings with those in humans. The implication of the human gut microbiome in the development of multi-morbidity symptoms will also be tested by microbiota transplantation experiments into rodents and phenotypic analyses.

#### b. Cellular models to identify causal molecular mechanisms of ELS-induced multi-morbidity

**Fig 5:**
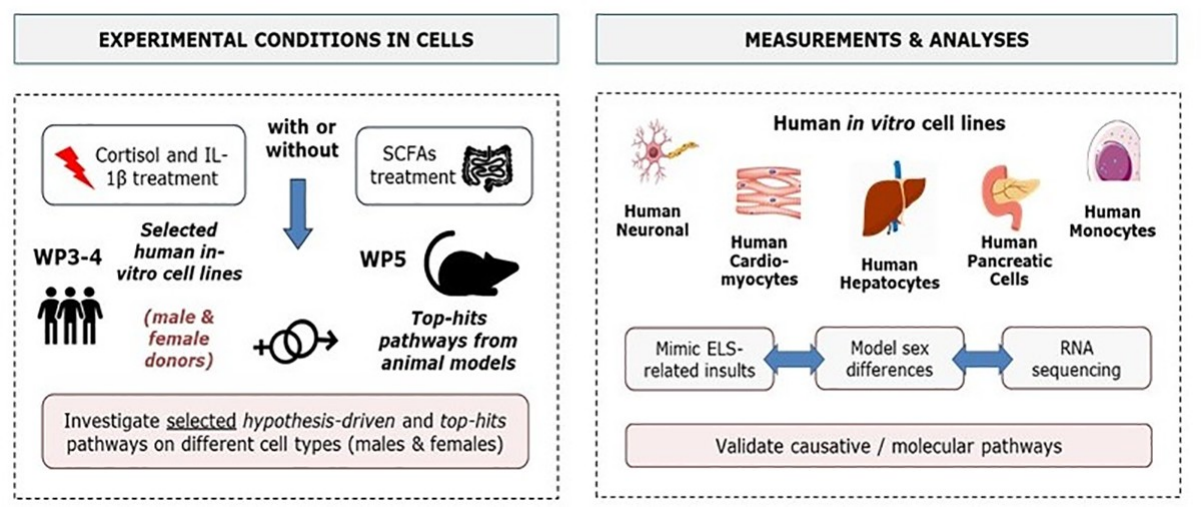
Overview of the cellular modelling experiences. SCFAs (Short-chain fatty acids).

We aim to uncover causative molecular and biological mechanisms underpinning ELS-associated multi-morbidity between depression, coronary heart disease and diabetes type 2 associated with ELS by leveraging a variety of human cellular models (*Fig 5*). Specifically, we aim to:

- Establish *in vitro* conditions to mimic stress and metabolic insults in human cell lines and primary cultures derived from brain, heart, liver, pancreas, and blood immune system;
- Study the effects of ELS on different cellular phenotypes related to depression, coronary heart disease, and diabetes type 2;
- Test causal mechanisms based on candidate biomarkers obtained from human and animal studies through molecular manipulations in selected cellular systems;
- Identify the molecular signatures of ELS in the distinct cellular types.

In particular, we will use a coherent, systematic approach to mimic ELS-relevant insults across a variety of cell lines and primary cells (from male and female donors), isolated from the human brain (hippocampal progenitor cells, hypothalamic neurons and cortical microglia), heart (cardiomyocytes), liver (hepatocytes), pancreas (pancreatic cells), and blood immune system (peripheral blood mononuclear cells), in order to identify the molecular processes induced by ELS that influence cellular and tissue homeostasis, and resulting in multi-morbid symptoms. For this purpose, cells will be exposed to candidate stress insults, including cortisol and interleukin-1 beta (IL-1β),and then both *hypothesis-driven* and *top-hits* mechanisms, that are confirmed across the human and animal models, will be investigated in each cell type (Table 3). Finally, mRNA sequencing analyses will be performed in the best 2–3 cell types, previously selected as the most representative of ELS-induced PCM multi-morbidity, in order to identify gene expression changes induced by treatment with stress (cortisol, IL-1β) on our selected cellular models.

**Table 3:** Biochemical and cellular candidate pathways that will be investigated in vitro.

Cell type	Biochemical pathways	Cellular phenotypes
Hippocampal precursors	Doublecortin (DCX), microtubule-associated protein 2 (MAP2), synaptophysin, Homer1	Neurogenesis; synaptogenesis
Hypothalamic neurons	Orthopedia, Neuropeptide Y, retina and anterior neural fold homeobox, ghrelin receptor	Differentiation
Primary cardiomyocytes	G Protein-Coupled Receptor Kinase 2 (GRK2)/cAMP; β-myosin heavy chain isoform; alpha-myosin	Number of myocytes and mature myocytes, energy metabolism
Primary hepatocytes	Phosphoenol pyruvate carboxykinase; glucose 6- phosphatase; glucokinase; pyruvate kinase	Gluconeogenesis; glycogen synthesis; glycolysis
Pancreatic EndoC- βH1	Dual Leucine Zipper Kinase; Beta cells transcription factors, PDX1, Nkx 6.1, MafA	C-peptide secretion, insulin secretion
Peripheral blood mononuclear cells	Interferon regulatory factors (IRF); JAK-STAT signalling	Immune activation relevant to atherosclerosis

#### 2.3 Computational bioinformatics and machine learning methods

We plan to implement advanced bioinformatic, statistical and machine learning techniques to integrate and leverage the findings and determinants derived from human studies and experimental models. Several types of integration will take place:

- Multi-omics integration of molecular interaction networks at different levels (DNA, RNA and proteins/metabolites) to dissect out the mechanistic chains across tissues;
- Structural equation modelling to model developmental timing and direction of associations, i.e. direct effects, as well as indirect pathways between variables and lifestyle factors affecting the pathways;
- Multi-cohort integration for bridging child/adolescent, adult and elderly cohorts and thus offer a life-course perspective on the link between ELS and multi-morbidity development;
- Machine learning models of multi-morbidity using unsupervised deep learning to simulate patient-specific trajectories towards multi-morbidity integrating identified biomarkers and pathways.

Subsequently, a proof-of-concept software will be assembled by integrating the predictive models within an existing e-health tool commercialised by COMBI; the Disease-State Fingerprint (DSF®). EarlyCause will extend it to account for multi-disease data and associations for the first time. The obtained tool will be pilot tested by COMBI’s usability experts to assess its acceptance and potential in future clinical management of multi-morbidities.

### 2.4 Centralised research platform

**Fig 6:**
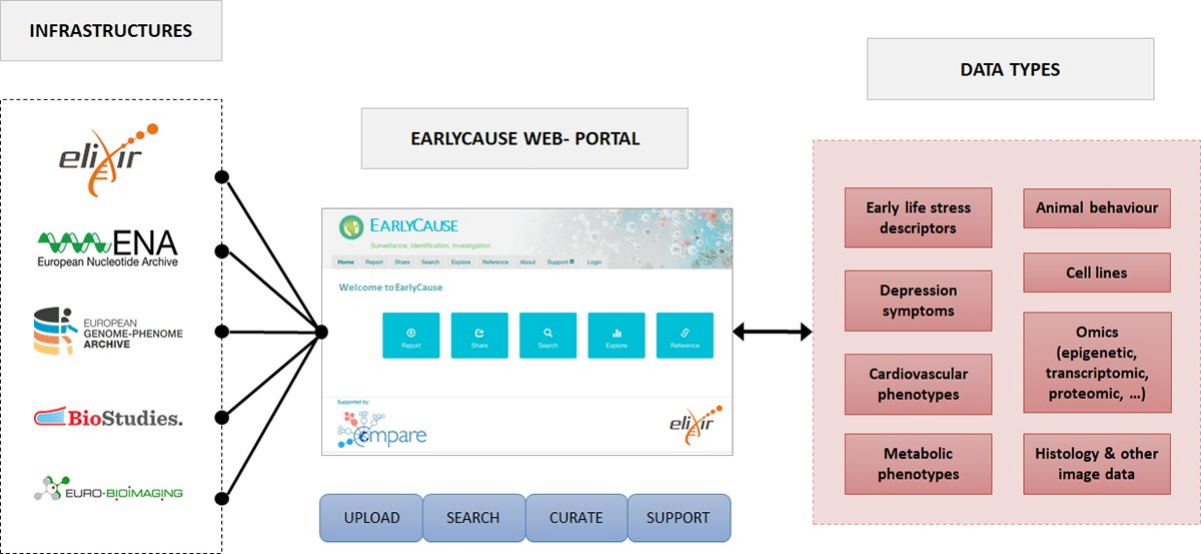
Overview of the EarlyCause centralised research platform, which will allow us to upload, search and manage human and experimental data for investigating ELS-induced multi-morbidity.

The research proposed in EarlyCause is novel, integrating causal inference studies, experimental models of both pre- and postnatal stress, and new computational approaches for uncovering the causal effects of ELS on multi-morbidity development. The expected results, including those on the role of epigenetics, microbiome and environmental modifiers, will set the stage for new studies to generate knowledge and contribute to public health guidelines. We aim to establish a research-enabling web-platform that will integrate data services, experimental standards and best practices to support next-generation research on ELS and multi-morbidity. The EarlyCause web-portal and centralised platform represented in *Fig 6* will provide a comprehensive support tool to researchers, which will allow them to upload and search data relating to ELS-induced multi-morbidity. For full FAIR (Findable, Accessible, Interoperable and Re-usable) compliance, our strategy is to build upon existing life-science/data infrastructures such as ELIXIR [34] and the EMBL European Bioinformatics Institute.

A key feature of the web-portal will be a rich environment for the discovery and selection of appropriate data sets and relevant project protocols for further exploration. These functions will leverage and adapt the existing data hub/portal software framework as developed and used in such projects as COMPARE [35] and HipScI [36]. The EarlyCause web-portal will be linked to ELIXIR’s “core” and “deposition” databases, notably the European Nucleotide Archive [37] (ENA) and European Genome-phenome Archive [38] (EGA) for fully open and controlled access molecular data, respectively, as well as BioSamples [39] for sample-related data, such as ELS exposure, rodent model stress descriptors, and Biostudies [40] for a variety of assay data types, such as rodents behavioural data and metabolic profiling. For image data, in particular rodents histology, we will leverage the image database from euro-BioImaging [41].

### 2.5 Impact assessment and exploitation planning

Since the study of ELS and its effects on multi-morbidity represents a novel research field, the EarlyCause consortium will perform a thorough impact evaluation, spanning socioeconomics, healthcare practice, prevention strategies, as well as technology and market analysis. The analysis will be built upon the ASSIST tool-kit, which will use quantitative input from literature- and expert-informed data, established socioeconomic models and Monte-Carlo simulation, to perform a qualitative analysis for different stakeholders, e.g. users, beneficiaries, payers, technologists, organisations, or health-systems. The experience obtained in the impact assessment of the C3-Cloud EU project [42], which developed clinical decision supports for the management of multimorbid chronic patients, will strengthen these activities. For healthcare practice, ex-ante scenarios will be designed for three countries (Germany, Spain and the UK) and compared to as-is situations to assess the potential impact of the research findings (new biomarkers, causal mechanisms, specific role of modifiers such as microbiome) on multi-morbidity screening and prevention. The resulting evidence-based impact assessment will contribute to the accelerated diffusion of project results and their acceptance by the social care, healthcare, and policy communities and facilitate future research activities.

### 2.6 Ethics and dissemination

The study has been approved by the Ethics Board of the European Commission. The results will be published in peer-reviewed academic journals, and disseminated to and communicated with clinicians, patient organisations and media.

## 3. Discussion

**Fig 7:**
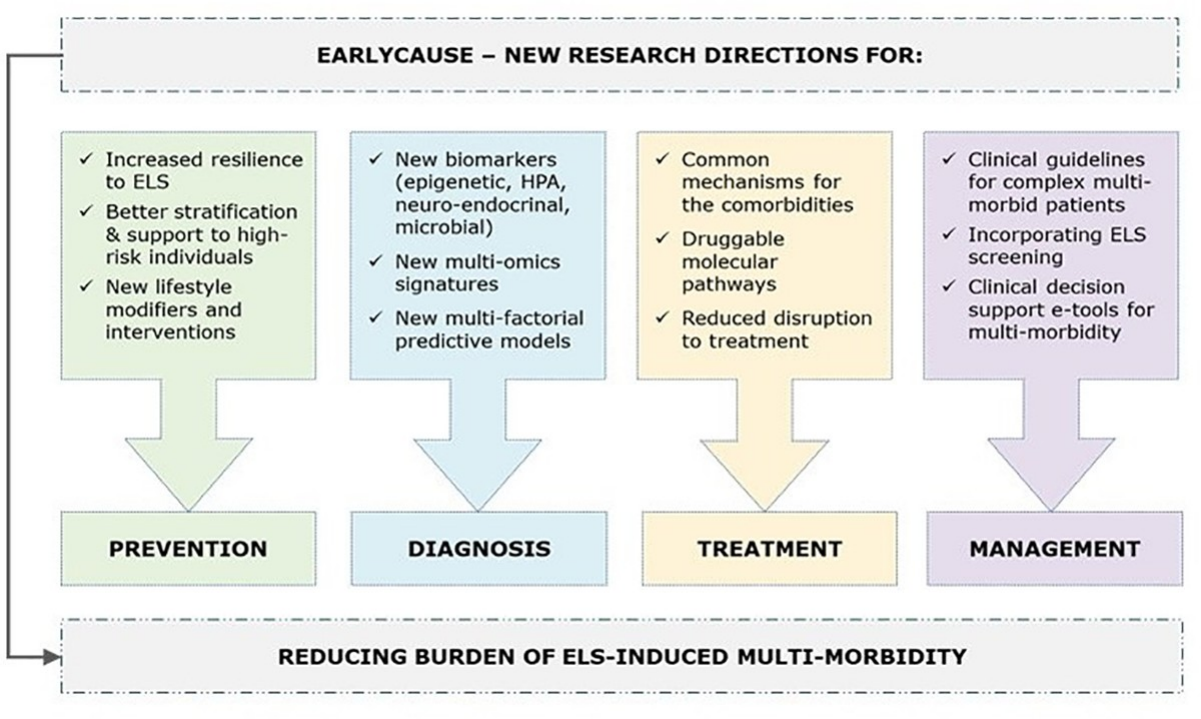
Overview of the research directions affected by EarlyCause.

Overall, EarlyCause will explore new territories at the interface of fundamental and clinical research by addressing the question of how ELS biologically impacts PCM multi-morbidity development. This will provide a rich series of translational research lines for targeting prevention, diagnosis, prognosis, therapy development, and management of PCM multi-morbidity (*Fig 7*).

### New directions for prevention and diagnosis of ELS-induced multi-morbidity

EarlyCause aims to create knowledge about the causal impact of ELS on multi-morbidity with the goal to inform the development of prevention programmes in two main directions. The first direction concerns the allocation of resources to schemes focused on reducing ELS per se, such as by providing greater support to high-risk families during pregnancy (e.g. midwife support, family and school-based programmes), or by increasing resilience to ELS through supporting early emotional, behavioural, and physical regulation in children. The second research direction concerns the identification of relevant targets for preventing multi-morbidity itself. Information on (the direction of) causality will allow the most effective primary preventative strategies to be established. This might focus on promoting lifestyle changes that affect possible shared causes of multi-morbidity, or treating the primary cause directly, or preventing/treating all multimorbid conditions together. In addition, knowledge of the role of ELS in PCM multi-morbidity development can also enhance the identification of multimorbid conditions in patients screened as having been exposed to ELS and who have already been diagnosed with one disorder (e.g. depression, but not yet diabetes or coronary heart disease). Furthermore, EarlyCause will combine ongoing research lines in a unique framework between ELS, inflammation, HPA, and microbiome, which will be scaled-up and extended to include different ‘omics’ levels (e.g. microbiome, genomics, epigenetics). This will open new routes to diagnose multi-morbidity beyond the simple addition of traditional symptom-based categories, promoting the development of a more biologically-informed nosology of multi-morbidity.

### Therapy development

EarlyCause will also promote new research for identifying targets for intervention. A natural next step will establish whether known drugs can impact the identified biomolecular pathways (so-called drug repurposing). This will open a host of potential future clinical trials using repurposed drugs that target these specific mechanisms. Randomised controlled trials are the gold standard for obtaining evidence on the effects of modifying disease risk processes. However, traditional drug (repurposing) development has several limitations, including short follow-up, small sample size, and non-representative samples. In this case, our Mendelian randomisation-based findings on PCM multi-morbidity can have direct implications for drug repurposing or the identification of unintended drug side effects.

### Management of multi-morbidity

Finally, knowledge gathered from EarlyCause will open opportunities for developing new patient pathways and care models for addressing ELS-related PCM multi-morbidity, complemented with an innovative set of technical solutions for improved clinical decision-making. The key aim of EarlyCause is also to identify lifestyle factors that dampen or exacerbate the impact of ELS on PCM multi-morbidity risk. Such knowledge will impact the implementation of lifestyle changes that can ameliorate symptoms and disease course, particularly amongst those who have already been exposed to ELS. EarlyCause will therefore improve existing clinical guideline recommendations with economic modelling of benefit and harm. Our ideal end-point will be to publish evidence to inform the future development of more streamlined and optimised multi-morbidity care pathways, thus improving decision-making and clinical management of patients with ELS-related PCM multi-morbidity.

## 4. Conclusion

In the coming years, EarlyCause will establish extensive research linking human, animal and cell studies with the aim to clarify how ELS biologically impacts PCM multi-morbidity development. The consortium will operate on FAIR data management and open science practices aiming to impact on the development of diagnostic and new health policies to alert clinicians on to the damaging effects of ELS and prevent its lifelong consequences.
